# Dental Anesthetics

**Published:** 1907-03-15

**Authors:** S. Straith


### DENTAL ANESTHETICS.

BY S. STRAITH D.D.S.

Read before the Detroit Dental Society, December 13, 1902

If we were to search for words in the English language that mean the most to the human race, we certainly would not over look the word *Anesthesia.*

Ever since the "deep sleep" fell upon Adam that he might, painlessly, be robbed of a rib, from which, as a nucleus, there was created for him, an helpmate, people have sought relief from pain in the use of agents, having real or supposed anesthetic properties.

The immeasurable blessings conferred upon mankind by Anesthesia can never be appreciated, and the homage due to the memory of its discoverer can never be paid.

To give the history of Anesthesia would take us back farther than the days of Davy, Wells, or Morton, to whom we usually give the credit for its discovery. However, this paper is not intended for that, but to give, as briefly as possible, the writers views of the relative merits of the Anesthetics more commonly used by the dentist, supplemented by the views of others both at home and abroad.

While it is well known locally at least, that the writer in his practice has a choice of Anesthetics, yet in dealing with this subject, it shall be the aim to give views unprejudiced by personal opinion. It is also his constant aim to be so unbiased, that conviction and conversion shall not be hindered, upon proof of merit.

We, as dentists, have to deal with a greater aggregate of pain, than the M. D.'s. This being true we should be doubly interested in the relief of pain.

When a patient seeks the services of a dentist, it is to preserve the usefulness of his teeth or because of endured, or to ward off expected pain, and uppermost of all in the patient's mind is the hope of painless relief from actual or anticipated suffering.

Whether it be for the extraction of a tooth, the filling of a cavity or the making of a plate, the dentist is consulted because of his special preparation and supposed fitness for that work; and he is expected to give his patient as little discomfort as possible, consistent with thorough work.

If. then, the dentist's services are sought because of his superior fitness for that work, why should he be instructed by the patient as to the material used or the manner in which it is to be used.

There was a time when the dentist would scarcely presume to count himself or his profession on a par with the M. D. or his profession; but to-day. while we have lost none of our admiration or respect for the medical profession, we have learned to respect our own profession in a degree more becoming its usefulness; but with this increased respect for ourselves we have yet failed to do our part in educating the laity to respect us as would become them, which respect the M. D. readily commands.

What would you think of yourself, should you after consulting your physician about some real or imaginary illness, begin to dictate to him what remedies he should use in treating your case? What do you imagine the physician who respects himself, would say to you?

This condition of affairs is a daily occurrence in the vast majority of our offices, and we docile creatures never disabuse our patient's mind of the impropriety of his actions.

When a patient presents himself to you for special services, he is not supposed to know, *and ought not to be allowed to think he knows*, whether a cement, amalgam, gold or inlay filling, or a crown is best suited to his needs. This is as truly applicable to the administration of ANESTHETICS as it is to any other work of the dentist;—and why is it not much more so? The former deals with the life of a *tooth*, the value of which is too often underestimated but the latter deals with the life of the *patient*.

We fall short of our duty to our profession when we allow opportunities pass to impress our patients with the belief that *we*, and not *they*, are the rightful judges of methods and materials indicated for each duty we have to perform. There are among us, dentists who have patients that, when applying for services, simply say Dr.——"Please fix my teeth"; and when the work is completed they know for the first time, the kind of work for which they are to pay. This is reasonable and just, and reposes confidence where it belongs; and the dentist that betrays this confidence by using methods or materials, which, to the best of his knowledge, are either contra-indicated, unnecessary or unfitted for the use to which they are put, is unworthy of the title possessed and forfeits the respect of his fellow practitioners.

Concerted effort upon our part will hasten the day when patients will place themselves as unreservedly in the hands of their dentist, as they now do in the care of their physician.

In considering anesthetics we shall refer to their use in extracting only.

When a patient presents himself for the extraction of a tooth, our first duty to the patient is to determine if extraction is indicated; and if it be, then the *choice* of an Anesthetic is the *next* thing indicated; and every time we ask the patient what Anesthetic we shall use, or allow him to dictate, just so often do we lower our professional dignity, admit our incompetency, and invite lessened respect for our ability from the patient.

If you were to submit to an operation for the amputation of an arm would your surgeon ask you what Anesthetic he should use, or would he acquiesce in your demands or requests for one contra-indicated or in the use of which he feels uncertain of success?

Then if, from this reasoning, we believe ourselves to be the right and *only* ones to select an Anesthetic, what shall govern our choice?

If we are uncertain of the element of the quality of an Anesthetic that should command our first thought, let us offer as a patient for extraction, our child, parent or friend, and with one accord the word SAFETY is indelibly photographed upon our minds. Next to safety we may consider, (2) Convenience, and, (3) expense.

If *safety* is to command our first thought, then the matter of Convenience or Expense should receive very little consideration.

What is it that shall decide this all-important question for us? We must either rely upon statistics, or *experiment.*

We know that Anesthesia is a *pathological* and not a *physiological* condition. From this we must observe that ABSOLUTE SAFETY in the use of *any* Anesthetic does not exist; for, where there is a pathological condition there is danger.

In the early days of Anesthesia, experimenting was quite necessary, and the surgeon could be more readily excused for failures or accidents. "But," in the language of Lyman in his book on Artificial Anesthesia, "for a large number of anesthetic substances, the age of experiment has passed. Their qualities have been ascertained by innumerable trials. To continue, then, to employ certain articles without restriction can only be deemed unjustifiable and culpable."

If then, we are not justified in experimenting with new untried or unproved anesthetics, we are to decide first, the safety of those that have been tried.

Authorities are almost a unit in the opinion that Chloroform and Ether have no place as an anesthetic in Dental practice, unless in aggravated cases, where the safer ones have failed, and then the administration should be intrusted to a physician whose practice necessarily better fits him to assume the responsibility of their administration. It is true that we, as dentists, are legally permitted to administer any anesthetic; but, all things that are lawful may not be expedient.

For the purpose of comparison, we will consider the four anesthetics most commonly used by the dentist. $N_2O$, Ethyl Chloride, Somnoform, and Local Anesthetics.

We are all very prone to tell of our successes, but few of us flaunt our failures or accidents before the eyes or ears of the public; so that it is very difficult to compile a list of accidents or deaths from Anesthesia. To assist me in the preparation of this paper I wrote a letter to 25 different dental colleges in the U. S. and to about the same number of colleges in foreign countries, asking several questions concerning Anesthetics. The replies received were not as numerous as I hoped for but they give an index of the opinions held by many men entrusted with the teaching and administration of Anesthetics in the educational centers.

The following are the questions asked:

(1) Which of the following is the most used in your college, or locality and why?

(a) Nitrous Oxide.

(b) Ethyl Chloride.

(c) Somnoform. (Information particularly desired.)

(d) Local Anesthetic.

(2) What are the objections to those least used?

(3) If you use Local Anesthesia, what formulæ?

(4) What have been the TOXIC effects of any one of them.

Any suggestions or informations will be gladly received.

In acknowledging these replies, we give every man credit for a conscientious, unbiased opinion; and when we do this, if we are to accept the decision of these leaders of thought, as our guide, and are to be governed by the majority, the dentist has only ONE choice.

We find that from the view of safety, $N_2O$ has no equal and none that can compare with it. It is estimated that in the U. S. alone, in the last 40 years, gas has been administered fully 1,000,000 times and about 8 or 10 deaths from its use are recorded. Out of this number only ONE patient died on the operating table. Of the others, 3 or 4 were asphyxiated by the lodgment of a tooth or other foreign substance in the respiratory tract and upon others, a post-mortem revealed conditions which would have contra-indicated the use of ANY anesthetic. In my search for for statistics, I fail to find the record of a single death in which gas has been charged as the direct cause.

Very soon after the discovery of gas, the anesthetic properties of Sulphuric Ether were discovered and following closely upon that, the discovery of Chloroform.

Upon the advent of these anesthetics the use of $N_2O$ for continued anesthesia was soon discarded because of the cumbersome apparatus necessary for its manufacture and administration,—In those days liquid gas was unknown. I have no doubt also that the means of manufacture made it quite impossible to get gas as pure as we get it today, and hence a less satisfactory anesthesia than that produced by the use of the strongest anesthetics—Ether and Chloroform.

The opinion formed of gas at that time as to its transient or uncertain effects has seemed to cling to the medical profession ever since; and it occurs to me that it has not tried very hard to disabuse its mind of the fallacy of the opinion. However, the frequent deaths from the use of Chloroform have led many physicians in the last 10 years to resort to the use of gas anesthesia,—particularly in cases where the use of Chloroform or Ether were plainly contra-indicated. In each case the anesthesia was quite satisfactory. Now if $N_2O$ will produce satisfactory anesthesia in cases where the risk is too great to use Ether or Chloroform, why should it not be used where such risks do not attend the use of an Anesthetic? This is unfair to gas; for, if a death should occur in such a case, that death would count just as much against gas as one occurring where no contra-indications were apparent. Every anesthetic has its limitations, but no doubt a very large number of anesthesias under Chlo. or Ether might be conceded to Nitrous Oxide with lessened danger to the patient. In the last few years the use of $N_2O$ and oxygen, either pure, or as contained in atmosphere air, is taking the place of the more dangerous anesthetics for many operations and anesthesia, lasting from ½ to 2½ hours has been obtained to the complete satisfaction of all concerned. The writer has had no experience with $N_2O$ and pure oxygen; but, out of nearly 5000 administrations of gas since coming to Detroit 3½ years ago, about 300 have been for surgical work,—minor and major, with the anesthesia lasting from one minute to twenty minutes. In each case the anesthesia was as long as desired, and could have been prolonged indefinitely. The patients ranged in ages, from 2 years to 84 years, and the operations, from the incisions of an abscess, to the removal of a tumor from the breast or the removal of an eye. The last case mentioned was that of a lady 71 years of age, in which the use of Chloroform or Ether was plainly contra-indicated and would not be given.

$N_2O$ is very commonly used now in the hospitals as preliminary to Ether Anesthesia, with the result that nausea so commonly accompanying the administration of Ether, scarcely ever occurs. It is also used with Ethyl Chloride in diffirent percentages throughout the anesthesia as will be noticed by one of the reports from foreign colleges.

Ethyl Chloride seems to be in favor in some localities, when a littlt longer anesthesia than that of $N_2O$ is desired, and is used in preference to Ether or Chloroform because of the lessened danger. Its physiological effects arc quite similar to those of Ether.

While it is considered quite safe, yet we find that at least 23 cases of fatality from its use are recorded within the last 3 years, by Dr. Luke, Edinburgh, Scotland.

Out of this number 8 cases were for dental operations. Dr. McCardie of Birmingham, Eng. reports 35 deaths, 17 of which were dental cases.

It is generally conceded to be safer than cocain, and the special study given its administration in recent years has revived its use, and results obtained warrant further investigation and trial.

Somnoform sprang into the lime-light a few years ago and from the way the bait was gobbled up, one would believe that the promoter of the anesthetic was a good fisherman; for he certainly found the wind in the right direction to make the fish bite. The commercial product has surely been a bonanza for its promoter, if it has not been a blessing to mankind.

I have always believed it better to be on the conservative side in the use of new remedies, and my conservatism at times, has caused the stamp of bigotry to be applied to me by some of the profession. This term was applied to me when I refused to use Somnoform. Finally one of our members said to me,—"Dr., you do nothing but administer anesthetics, and we look to you to try out these new things and give us the benefit of your experience."

At first thought that sounded logical and it occurred to me that possibly I was standing in my own light, in not using an anesthetic that was proving such a boon to the dentist.

I procured an outfit at once and proceeded to use it,—when the patient objected to gas, or where the need of a longer anesthesia than that of $N_2O$ was apparent; for this is one of the claims made for Somnoform,—a longer anesthesia. I gave the anesthetic 6 or 8 times with success varying, from good to ordinary. Finally, symptoms in 2 or 3 cases which, in themselves, *may* not have meant anything, yet they were new to me, awoke me from my dream, and I concluded that I had no right, legal, professional, or otherwise to experiment with uncertain remedies and thereby jeopardize the lives of patients entrusted to me and discarded its use.

In discussing this subject at a meeting of the State Dental Society, two years ago, I was quoted in one of the circulars distributed by the promoters of Somnoform as saying that, "Dentists should try new products," which is directly opposite of what I did say, and what I still believe which is:- new products should be tried in the colleges and passed upon before they are indiscriminately used by the general practitioner, who is less able to cope with unfavorable conditions which may arise from their use.

Through the kindness of our president, I was referred to an article in the October number of the *Dental Review* on Somnoform, written by J. W. Ritter, D.D.S., Charleston, Ill. If there are any of you who are using Somnoform or contemplating its use, who have not read the article you certainly should not fail to read it.

I shall refrain from further comment upon this subject, believing that the information given you in the letters received in reply to my questions sent to the colleges will prove more helpful to you than anything I could say.

Local anesthesia is certainly a great boon to humanity; and while, no doubt, its use is often abused, yet it has proved itself worthy of a place in the surgeons' pharmacopoea.

The uses to which local anesthesia is put are varied and numerous, and the pain alleviated, or avoided by its use will never be appreciated.

Local anesthesia had been practiced for many years before the advent of cocain, which is the active principle in eight tenths of the preparations now used.

The wonderful effects of cocain and the varied purposes it might subserve soon became very apparent and its indiscriminate use was followed by troubles which, becoming known, made the profession much more cautious in its use. Numerous articles appearing in print from time to time relating troubles with the drug, serious or otherwise, advised the laity of the dangers accompanying its use. To over come the prejudice caused by these printed articles, and to still retain the demand for local anesthesia, the fertile mind of the dentist, or the commercialist, brought forth "Alvatunder," "Odontunder," and many other preparations for local anesthesia. These names were given to the mixtures to divert the attention of the laity, and some unsuspecting members of the profession from cocain, because of the prejudice caused by unfavorable results arising from its use.

Chemical analyses, however, show percentages of $\frac{3}{4}\%$ to $2\%$ or more of cocain, in most of these preparations.

It is quite safe to say that there are more teeth extracted every day under local anesthesia, in our country, than under any other anesthetic; and possibly more than all other anesthetics combined.

The dangers attendant upon the too careless or promiscuous use of local anesthetics are so commonly known and experienced that they need not be elaborated in this paper.

With some of the more cautious dentists, Eucaine B is being used, in $2\%$ solutions, to the exclusion of Cocain. It is said to be only about $\frac{1}{8}$ as toxic as cocain, but is liable to be followed by more swelling.

In discussing any of these anesthetics, it did not occur to me necessary, or within the bounds of this paper to speak in detail of the physiological effects, or the manner of their administration, as much of this will no doubt be brought out in the discussion of the paper.

The convenience and Expense of the several kinds of anesthetics will be briefly considered, together.

When we consider Convenience to include the initial expense of an outfit and ease of conveyance or manipulation, then there is nothing to compare with local anesthetics; but if we include in the item of expense, the dentist's time, we soon find that Ethyl Chloride or Somnoform will demand first place.

If two or more teeth are to be extracted, there will have been time to administer any one of the other anesthetics, extract the teeth and dismiss the patient before local anesthesia could be produced.

In the reading of the letters received in reply to my questions, you will notice that, with the exception of one convenience or lack of anesthesia is the only reason for not not using $N_2O$.

As stated before in this paper, the writer believes that these factors should receive very little consideration.

LETTERS.

(We omit dates and forms to save space.—ED.)

1. Nitrous Oxide as a general anesthetic for minor operations.

2. We use Ethyl Chloride only as a refrigerant or obtundent.

3. Somnoform we exclude entirely, as we do not feel it is proven to be as safe an anesthetic as Nitrous Oxide.

4. We use P. D. & Co. tablet No. 81 (Dr Hoff's formulæ) and No. 151 for local anesthesia. 81 is:

Cocain hydrochlorate, ½ gr.

Morphine sulphate, - ⅛gr.

Atropine sulphate, 1-200 gr.

We do not administer percent solutions of these drugs, but use definite dosage in parts of a grain.

5. We have no other than an occasional nausea. Have had no serious effects from the use of cocain preparations, but they are used under the greatest precautions, absolute cleanliness is observed in each administration.

Formulæ 151 is P. D. & Co. adrenalin 1-300 gr. and cocain 1-6 grain, and is used when an anesthetic is needed and also when we wish to control hemorrhage.

Thos. L. Gilmer, M.D., D.D.S.

*Northwestern University.*

1. Nitrous Oxide.

2. No great objections. Ethyl Chloride in the hands of students may cause degeneration of tissue, is not readily controlled and is expensive. We use Somnoform quite a good deal but do not consider it has proven its position in therapeutics to the extent Nitrous Oxide has. Also use Local Anesthetic.

3. Until recently we used solutions of Nirvanin because we believed we had less toxic effects but are now using cocain hydrochlorate as follows:-

| | |
|---|---|
| Cocain hydrochlorate, | grs. iv. |
| Morphin, hydrochlor., | grs. 1¼. |
| Nitro-glycerine, 1 to 100, | m. xv. |
| Carbolic acid, - - - | m. viii. |
| Distilled water to make - | ℥j |

Geo. E. Hunt, D. D.S.
*Indiana Dental College.*

---

1. Nitrous Oxide because it has given us on the whole the most satisfactory results and is practically free from unpleasant after effects.

Ethyl Chloride is however, rapidly coming to the front as an introductory anesthetic. Probably this is due to the fact that better appliances are used and more experience is had. Some good authorities claim that it may replace Nitrous Oxide.

Two years ago Somnoform was used almost exclusively in our clinics with only two cases of unfavorable symptoms. Somnoform in well selected cases is certainly an ideal anesthetic for short operations; but it cannot be used nor should its administration be advised in every case like Nitrous Oxide.

We had two unfavorable cases, one a patient suffering with peptic ulcer, and the other a patient addicted to the use of acetanilid powders.

Of local anesthetics we use cocain in a 1% solution for extractions and in 3% sol. for removing pulps.

With Nitrous Oxide we have had no unpleasant symptoms, but have had some cases where it was not successful, while Somnoform used in the same case gave the desired results.

E. T. Loeffler, B.S.,D.D.S.
*Michigan University.*

---

We use Nitrous Oxide *only* because it is well tried and safe.

E. C. Kirk, D.D.S.,
*University of Pennsylvania.*

---

We use Nitrous Oxide exclusively.

M. C. Marshall, D.D.S.,
*St. Louis Dental College.*

---

Nitrous Oxide is used almost to the exclusion of general anesthetics in our college extracting room; the number of extractions under an anesthetic amounting to 1412 in one year. This would include perhaps fifty administrations of Somnoform and 250 administrations of a local anesthetic hypodermically.

We avoid the use of Ether and Chloroform and their mixtures in dental operations because we consider Nitrous Oxide sufficiently satisfactory and because we feel that we are not justified in assuming the risks which are connected with the administration of Chloroform and Ether in minor operations; and I think this opinion is entirely concurred in by surgeons and by Hewitt in his recent text-book on Anesthesia. A familiarity with Ether and Chloroform administration is obtained through witnessing operations in clinical oral surgery.

Regarding Toxic effects from Nitrous Oxide they are practically nil, and we may say the same of Somnoform, so far as it has been used. We are of opinion that untoward after effects are more common in cocain anesthesia than from Nitrous Oxide.

We believe Somnoform is more satisfactory than Ethyl Chloride has been, but should be accepted with caution and that it will never probably be so good an anesthetic for the Dentist's use as Nitrous Oxide.

In this connection we might call your attention to a statement recently made and which you have probably seen that "Nitrous Oxide is safer than crossing a crowded street."

H. T. Smith, D.D.S.,
*Ohio College of Dental Surgery.*

---

We use mostly Nitrous Oxide.

Somnoform, used for three years in this school and found to be safe thus far. Local anesthetic:

| | |
|---|---|
| Morphine sulphate, - | gr. $\frac{1}{3}$. |
| Cocain muriat, - - | grs. $3\frac{1}{2}$. |
| Listerine, - - - - | oz. $\frac{1}{4}$. |
| Dist. water, - - - - | oz. $\frac{3}{4}$. |
| Sodium chloride, - - | dr. 1. |

M. W. Foster, D.D.S.
*Baltimore College of Dental Surgery.*

---

We use general and local anesthesia. In general anesthesia we prefer Somnoform to $N_2O$. For local anesthesia we use Ethyl Chloride spray, rarely cocain solution injection. Somnoform gives a longer period of anesthesia with shorter period of induction. It is safer and more pleasant than $N_2O$ and the patient remains quiescent throughout the operation.

H. A. Nolan, D.D.S.,
*Supt. of Infirmary.*
*New York College of Dental & Oral Surgery.*

We use Nitrous Oxide, Somnoform and Local Anesthetics about equally, and do not have any particular choice except as the case may present a history that would contra-indicate any one of them. Even though we might have a choice, in college work it is advisable to teach the use of all, giving more prominence to the one most generally used.

We do not use Ethyl Chloride in the clinics so can not give data except from my own experience.

The local anesthetic used in the college is one having Potassium Iodide, Tinctures Aconite and Iodine, Glycerine, Carbolic Acid, Aquæ Cinnamon, Distilled Water. We find one containing cocain more or less dangerous in the students hands.

We have had no trouble in the last three years; but have had one death from Nitrous Oxide, due to shock following. Have had two or three cases of Cocain poisoning.

K. C. Brashear, M.D.,D.D.S.,
*Ohio Medical University.*

---

The anesthetic most used in our infirmary is Nitrous Oxide Gas, because it is reliable for short operations and has proved itself the safest of all anesthetics. We use Chloride of Ethyl locally as a spray and find it very useful in many cases. We do not give Chloride of Ethyl by inhalation. We have no objection to mention against such use, but it has so happened that we have never felt the need of trying it.

Have used Somnoform more or less for a year and a half. It does not. however supplant to a very great extent the use of Nitrous Oxide Gas. It is favorably regarded by those who have administered it. While no serious symptoms have been shown in its use at the Dental School, yet all feel that being a comparatively new agent special care should be exercised in its administration.

As to local anesthesia we employ as before stated Ethyl Chloride as a refrigerating agent and also Cocain Hydrochlorate by hypodermic injection.

In the use of cocain for injection we consider that 1-8 of a grain is a maximum dose.

The use of adrenalin in connection with cocain is favorably regarded.

William H. Potter D.M.D.
*Harvard University.*

---

We have used Nitrous Oxide ONLY for the last thirty years with out any dangerous symptoms.

Local Anesthetics not used at all.

The absolute safety of Nitrous Oxide precludes the necessity of experimenting with other agents general or local.

Faneuil D. Weiss, M.D.
*New York College of Dentistry.*

---

We use Nitrous Oxide almost exclusively as a general anesthetic and consider it the best anesthetic for our needs where the operations will only take from five to twenty-five minutes, as the work can be done painlessly and safely and accomplish all you wish to do and have so few disagreeable after effects. We use the nasal Hurd inhaler.

Ethyl Chloride we use as a local in the form of a spray but not as a general anesthetic.

Have used Somnoform some but the working period is too short and when attempting to prolong anesthetic cause too much sickness and nausea. It is very useful for children and short operations.

As a local anesthetic use a 2% solution of Beta Eucaine. Have used it for five years with very good results. Allow each member of the Senior class to use it freely for the extracting of teeth. Have never observed any toxic symptoms resulting from the use of this solution, and its anesthetic properties are very marked.

We did use Cocain in 1½% solutions, but in treating the number of patients we do, there would be some who would develop very alarming symptoms. And finally one case proved fatal. So we changed to Eucaine and have had no further trouble.

J. A Bullard, D.D.S.,
*Chicago College of Dental Surgery.*

---

Nitrous Oxide is still our favorite, it being considered the safest, and the results such as not to justify a change to newer and more dangerous methods.

Ethyl Chloride is not used by us although I use it successfully in the operation of tonsillotomy, though you do not get complete general anesthesia always you get more or less local effect.

Somnoform has been used often but having had alarming symptoms, we have discontinued it. Personally I have used it but twice. Case No. 1 was successful and normal. Case No. 2 took it badly, turned very blue and struggled violently and was much nauseated afterwards. Might state that the patient had cleft palate and was very nervous. One bulb of Somnoform was used.

For Local Anesthesia.—Local Anesthetic Tablets are used, i. e. Cocain Hydrochlorate gr. ½ Morph. Sulp. gr. ⅛, Atropine Sulp. gr. 1-200.

Jas. W. Pegram, M.D.
*Dental Department Milwaukee Med. College.*

---

1. In ordinary extractions in the Infirmary we use local anesthetic mostly. Where a number of teeth are to be removed we use general anesthesia by Ether, or Ether preceded by Ethyl Chloride, always administered by a physician.

(a) Nitrous Oxide is less used because the extraction of the common broken-off teeth and weakened fangs, by students, requires more time than Nitrous Oxide permits.

(b) Ethyl Chloride locally deserves to be used more than is our custom, as it is devoid of the danger that cocain presents.

(c) Somnoform not used by us for several reasons:

First, it is a commercial article, whose composition may be varied by the manufacturer at will.

Second, we have yet to learn a single point of superiority over the simple, pure Ethyl Chloride; we believe that both the name and the distinctive composition of Somnoform serve commercial ends only. We feel it is not entitled to our consideration for the above reasons.

Third, the reported addition of Ethyl Chloride and Ethyl Bromide cannot add to its safety, as the bromide at least is more depressing. We should expect it to be more dangerous than pure Ethyl Chloride.

Fourth, it is more accurate and contributes more to our knowledge of these newer agents, to use them singly, so as to know the action of each separately. Ethyl Chloride has been shown to be comparatively safe. A longer experience with it is needed to establish its place absolutely. We ought not to allow ourselves to be diverted toward a proprietary, commercial preparation, that posses no superiority and whose composition probably makes it more dangerous.

3. The formulæ of Local Anesthetic mostly used is P. D. & Co's. No. 81. It is used in a dilution that represents ½% of cocain. We have had no bad results with this but it must be said that we are not partial to this particular formulæ; in fact, we do not care for the Morphine present as it has no local analgesic action.

Eli H. Long, M.D.

*University of Buffalo Dental Dept.*

I have been a strong advocate of Nitrous Oxide gas as a dental anesthetic for the past twenty five years, and up to the present writing I have no thought of or occasion for changing my mind, having administered gas over eighty thousand times without a fatality. I can name eight or ten specialists whose aggregate of administrations would amount to over six hundred thousand without a death.

Such a record has never been attained with any other anesthetic and to-day Nitrous Oxide is in greater favor and used far more by the medical profession, as well as by the dental profession, than ever before.

Other anesthetics have been introduced but instead of growing in favor have been discarded because of unsatisfactory results and risk to human life.

Judging from present records and Journal accounts Somnoform will sooner or later be discarded for the same reasons.

L. W. Nevius, D.D.S.
*Extracting Specialist,* Chicago.

---

By far the most commonly used general Anesthetic, for dental purpose in this country is Nitrous Oxide. It s considered very much the safest, and only in cases which require a longer time than the anesthesia of this agent ordinarily lasts, are the other general anesthetics used. Among dentists in this city, Ethyl Chloride and Somnoform are scarcely used at all. Any clinics that have been publicly given with these agents have been unsatisfactory. The very general consensus of opinion in England against them also discourages their use here. Probably 75% of dentists use local anesthetics, many of them P. D. & Co'. tablets, and others one or other of the proprietary nostrums whose formulæ are unknown. At the college we use Nitrous

Oxide and Stovain. I am afraid of cocain and do not use it at all. Many of the practitioners have had more or less unpleasant experiences with it.

J. B. Willmott,
*Royal College of Dental Surgeons.*
Toronto, Ontario.

---

(a) Nitrous Oxide very little used now.

(b) Ethyl Chloride as general, (not as local) not much used.

(c) Somnoform and Coryloforme, which is practically the same thing as Somnoform, but cheaper are used to a great extent in preference to Nitrous Oxide, the anesthesia persisting longer than gas, the apparatus being smaller, and the liquid being in a tube sealed which may be preserved any length of time, and the quantity we are using being determinate.

(d) Cocain, 2%. Ethyl Bromide is also used by a good many practitioners, and I have used it extensively myself, but patients have nausea afterwards.

The information here given not only concerns our school but the practitioners in general in this province.

Dr. Eudore Dubeau,
*Dean La val Univ. School of Dental Sur.*
Montreal, Canada.

---

In private practice I use Nitrous Oxide almost exclusively, because the operations to be performed are nearly all such as require only a short anesthesia, for which purpose I consider Nitrous Oxide the best anesthetic, on account of its safety and practical absence of after effects. If there happens to be morc to do than can *easily* be done with one dose, I prefer to have the patient come back another day to have the operation completed. Occasionally however I find it necessary to use Nitrous Oxide and Ether, or Somnoform, but not often.

In hospital work I use it for short operations for the reasons given above, but the great majority of the cases have a great many roots to extract, and in these cases, time being a consideration, I use either Nitrous Oxide and Ether or Somnoform, probably the one as often as the other. I use Nitrous Oxide and Ether in all weak anæmic cases, mitral cases and so on, when a cardiac stimulant seems indicated; and Somnoform for ordinary straightforward cases, with nothing particular the matter with them.

All my cases are prepared for an anesthetic-except when Nitrous Oxide alone is to be given, and in consequence I have very little trouble during the anesthesia and after effects are not very common, the commonest being sickness and sometimes headache, after Somnoform especially.

I nearly always give Somnoform to men lying down in hospital. I found they struggled so when coming round in the chair, they seem to take it much better on a table. The hospital class of men in this part of the world all drink more or less, and rather more than less.

I don't know why I use Somnoform in preference to Ethyl Chloride, but I haven't a great deal of experience with Ethyl Chloride alone, and the few cases I have had didn't seem to be so satisfactory as Somnoform.

Local anesthetics I hardly use at all. I have found them anything but satisfactory except in the most trivial cases.

I am afraid this is a rather rambling letter, but I hope you will be able to extract from it the information you require.

J. Crombie, L.D.S.
*University of Aberdeen,* Scotland.

---

We use Nitrous Oxide gas in all cases except those in which a long time is required, then we use Ether.

Do not use Ethyl Chloride because of vomiting, and it is also considered dangerous.

Somnoform is not used at all.

Do not use local anesthetics.

We find no objections to Nitrous Oxide gas and like it best.

We have used Ethyl Chloride, Somnoform, and Local Anesthetics and found in them nothing to recommend them as against $N_2O$ gas.

E. A. Beavers, J.P.,M.R.C.S.
*Radcliff Infirmary,*
Oxford, England.

---

Nitrous Oxide is by far the most used by us in fact it is the routine anesthetic for dental anesthesia,- *because* it is by far the safest and has practically no after effects. For long cases, we use Pattersons apparatus for the nasal administration.

Ethyl Chloride is less safe than Nitrous Oxide. Nearly half the deaths in Great Britain under Ethyl Chloride have been in dental cases (17 out of about 35). and after effects much greater and more persistent.

Somnoform has no advantage over Ethyl Chloride, probably greater effect, certainly rather less safe and more liable to decompose, because of the Ethyl Bromide in it.

Local anesthesia not much used, though rather increasing, so I think. Objections, probably less safe than gas, special precautions during recovery, and much longer time from start to finish. Chiefly suitable for few and front teeth. Technique must be perfect and patient a grown up.

Ethyl Chloride and Somnoform cause heart and respiratory depression in big doses and occasionally prolonged after vomiting, even after small doses. Headache afterwards may be severe.

Local anesthetics are not used at all in the dental hospital and not much in private practice, and then only for extraction of one or two teeth which are in the front of the mouth. Waite's anesthetic solution is most used, although $\frac{1}{2}\%$ and $1\%$ solutions of Eucaine or Cocain with a little adrenaline are becoming more popular, I think.

W. J. McCardie, B.A.,M.B.B.C.
Anesthetist to the *Birmingham, Eng. Dental College.*

---

In the dental clinics I use either of the following without any prejudice.

For general anesthesia, Ethyl Chloride, Coryloform, or Somnoform.

For local anesthesia, Cocain or Stovain.

I guide myself in the employment of an anesthetic by the condition of the patient. I reject all general anesthetics for patients who have chronic organic diseases or lesions, or subjects who say that they have diseases that would contra-indicate the use of a general anesthetic.

I do not use general anesthesia with people who are very nervous, hysterical or epileptic.

I give cocain or stovain when people ask for them, except where there is inflamed tissue.

In all cases where general anesthetics are rejected, I use blue light to deaden pain, and am well pleased with it.

We use Nitrous Oxide very little in France. It does not come in conflict with the use of any other anesthetic.

Dr. Cavalie,
Bordeaux, France.

---

We have only a few trials with Somnoform. For the past five years, for extractions as well as for surgical interferences, we have used local anesthesia and have gotten so that we scarcely ever think of the narcosis.

As a local anesthetic we did use Adralgin, Alypin, Stovain but now we use $2\%$ solution of Novokain in connection, with a para kidney preparation, and this is of excellent service.

We have not noticed any poisonous effect, only in two cases among the many. We noticed Necrosis of a small portion of the mucous membrane around the point of injection.

Ethyl Chloride we use only rarely in making incisions.

Dr. Scheff,

*University of Vienna.*

DISCUSSION.

Dr. R. E. Loucks:-The subject has certainly been well and carefully handled by the essayist, and he deserves our sincere thanks for the pains he has taken to present so much evidence to us. There seems to be a considerable variety of opinion but this we should expect from the fact that men succeed best with the anesthetic they get best used to. Localities or conditions will many times control opinion. Older men will stick to Nitrous Oxide while the younger men will try out the newer remedies. There can be no question but Nitrous Oxide is the safest anesthetic, but yet it may not be the best one to use always or in every case. It may appear later that the fatalities with the newer anesthetics may be due to our inexperience in their use. We should say that Nitrous Oxide and Ether are the safest anesthetics for the novice, for Cyanosure stops us before we go too far with gas, and with Ether the patient stops breathing long before the heart stops beating. Patients should never dictate the form of Anesthesia, except that any bad experience they may have had should put the anesthetist on his guard. Every dentist ought to know what he can use best, and should be able to tell what agent his patient will take best, because of his greater skill with one agent than another, except when physical conditions control the choice.

The gravity or extent of operation should to a considerable extent control in the choice of the anesthetic. Age and physical condition of patient may sometimes change ones choice arbitrarily, as no exciting anesthetic like Nitrous Oxide or Ether should be given to epileptics or chronic alcolholics. In many such cases local anesthetics are effective and prejudice no unfavorable results. Some men are so skillful in the use of local anesthetics as to never need the general anesthesia. It is hardly fair for any one to say that any anesthetic will meet all cases, or that one is greatly superior to another. There may be cases where the most condemned anesthetic would produce admirable results.

Dr. Graham:-Individuality plays a larger place in the administration of anesthetics than in most any other department of practice. In giving Nitrous Oxide I have found that most dentists do not carry the anesthesia far enough. Dr. Straith gives all I should want my patient to take and then goes still farther with his anesthesia. Nitrous Oxide is an expensive anesthetic and the apparatus is cumbersome and calculated to frighten the patient. As a dental practitioner I do not feel that I ought to administer Ether or Chloroform although I may know as much of its physiological action as the majority of medical practitioners; but I can not know my patients physical condition so well as the medical practitioner, and therefore am not qualfied to use these powerful systemic remedies. In allowing chloroform or ether given in our offices we should know that the administrator is a skillful practitioner or decline the use of the drug.

Dr. M. L. Ward:-Nitrous Oxide is undoubtedly a safe general anesthetic, but in my judgment it should always be given in connection with oxygen, which will not only enhance its safety but also prevent its baneful secondary results. The Cyanotic symptoms are thereby prevented, the anesthesia is quiet and peaceful, and it may be prolonged to any limit that the operation requires with no shock to the patient. I have kept a patient in comfortable anesthesia for over an hour with no bad after results.

Dr. F. Shattuck:-I use a local anesthetic almost entirely and have never had a fatal or alarming result; and

I have used it for all classes of patients, old and young, fat and lean, nervous and lymphatic, as well as all kinds of inflammatory conditions of the mouth.

N. S. HOFF:-The paper of Dr. Straith and the replies of the men he has read to us, indicate that safety is the important end sought in all anesthetics. The question of degree of anesthesia does not seem to need discussion. And yet when we think of it there are more painless operations performed, or attempted at least, with no medicinal drug than where any or all known anesthetics are used. Dr. Straith, however is making an effort to establish the safety of Nitrous Oxide rather than the efficiency of it, and I believe he has made out a strong case in both qualities. There have been comparatively few deaths from the administration of Nitrous Oxide directly, but I am not sure that the list of fatalities might not be greatly lengthened if we could gather up the list of secondary results that this drug should be held responsible for. I have traced several cases of bronchitis to its use, and many bad colds have resulted either from the shock incident to its inhalation and lack of time for recuperation before passing out of doors in a cold winter air. I know of one case where a man took gas twice to have a large number of teeth extracted and then drove twenty miles to his home on a cold winter day, and within ten days died of pneumonia. I have no doubt that if the entire gas history were known we should not be so confident as to its virtues. As Dr. Ward suggests, I believe it should always be given with oxygen and that its asphyxiating tendencies should be avoided. Some patients can not be anesthetized with Nitrous Oxide.

I once assisted in giving a very fat man eighty gallons of gas with no anesthesia to speak of.

As to Somnoform I most cordially agree with Dr. Long of Buffalo, when he states that we should not use an article that is a commercial article, the composition of which may be varied at any time, and especially when that article is of such a dangerous tendency as this one. The drugs composing this compound are not uniformly stable and though the manufacturers may be ever so honest and capable the compound will not always be the same. This may account for the present high mortality of these compound anesthetics. The same objection can be made to the use of the local anesthetics. Besides all this where so many ingredients enter into these remedies it becomes difficult to diagnose and treat unfavorable symptoms as they may arise. One of the letters stated that its writer always used cocain solutions in dosage of definite parts of a grain regardless of percentage of solution. This we believe is a grave error. The dose of a high dilution solution may be larger without danger than when the solution is more concentrated and a smaller amount be administered. In other words a half grain of cocain diluted to one percent will not be as likely to produce toxic results as when used in a two percent solution, because the more it is diluted the less astringent effect it will have on the blood cells and consequently less liability to produce interference in the circulation and function of the nerve centers.

DR. A. L. LEGRO:-I have used somnoform ever since it was put on the market and have had no occasion to find fault with it, and I am not convinced by the essayist or discussion that I should abandon its use. I have observed no marked physiological aberations that might not occur with nitrous oxide or almost any other of the general anesthetics. Somnoform is not so irritating to the respiratory tract as the cold gas that comes from the liquidfied nitrous oxide: its chemical reaction with the tissue cells is more normally physical than nitrous oxide, which is merely a mechanical re-agent which is not decomposed in the body at all, consequently as soon as it is eliminated from the body the anesthesia ceases. You can have a longer anesthesia with somnoform.

DR. STRAITH:-I do not believe the average dentist should experiment with new remedies that are capable of so much harm to their patients. We should have confidence in the safety of our anesthetic and in our ability to properly administer it. I have given gas to every person who has asked for it, with a single exception. A patient with a very bad heart. I afterwards gave it to this lady on her physicians consent and with no bad result whatever. We should be able to make a clear physical diagnosis of every patient, but many times this is impracticable.

The time factor is not worth mentioning the half of a minute is not worth thinking about when other and more important factors are at stake. I make my own gas and know it is pure and I believe I get a better anesthesia than from the liquid gas. I use from four to six gallons where I used seven to ten of the liquid gas for each patient. It is highly important that the gas be given pure and with no air mixture as this will result in a subconscious state before and during the anesthesia. Air can be admitted after the anesthesia is secured. The objection that has been raised that the gas apparatus is cumbersome and unsightly can all be overcome by placing the gasometer in an adjacent room or closet and carrying the gas to the chair by a suitable tube.

